# Mental Flexibility and Epistemic Trust Through Implicit Social Learning – A Meta-Model of Change Processes in Psychotherapy With Personality Disorders

**DOI:** 10.32872/cpe.12433

**Published:** 2024-04-26

**Authors:** Svenja Taubner, Carla Sharp

**Affiliations:** 1 Institut für Psychosoziale Prävention, Universitätsklinikum Heidelberg, Heidelberg, Gemany; 2 University of Houston, Houston, TX, USA; 3 University of the Free State, Bloemfontein, South Africa; Philipps-University of Marburg, Marburg, Germany

**Keywords:** mentalization, mediated learning experiences, micro-process, corrective emotional experience, implicit learning

## Abstract

This position paper follows the call for transtheoretical meta-models of general clinical change by concentrating on severe mental illness such as Personality Disorders (PDs). We have identified a core process of change related to mental flexibility through implicit learning and propose recommendations for stance and technique that are informed by research on Mentalization-Based-Treatment (MBT) and the learning components as represented in the Mediational Intervention for Sensitizing Caregivers (MISC). While the idea of corrective emotional experience as a general change mechanism involves discriminating between an old and new relationship to update relationship knowledge, the capacity to understand and process corrective emotional experiences may be limited and even iatrogenic in patients with PDs. By integrating MBT and MISC, a meta-model of change is created that allows training in and observation of the granular-level, behaviorally anchored, actions taken by the therapist to open up social learning. Here, social learning is conceptualized as epistemic trust, increasing the client’s reflective functioning during sessions to ultimately enhance cognitive flexibility outside the therapy room. This opens the possibility to implement and observe micro changes in what should be termed now implicit cognitive and emotional corrective experiences. Thus, we propose to shift towards implicit learning within professional relationships; that is, internalizing a new way of thinking about any life-event that requires adaption thereby creating adaptive capacities via mental flexibility as the general change mechanism of Personality Disorder (PD) treatment.

## Highlights

- The paper provides a transtheoretical change mechanism for the treatment of Personality Disorders such as mental flexibility.
- Mediational learning experiences translate the model of corrective emotional experience in psychotherapy into a clearly defined process of implicit learning.
- Using the model of mental flexibility and mediated learning enables helping professions to establish new competences in managing helping relationships.
- For research on changes process, the model offers ways to investigate micro process in psychotherapy and helping professions.

The treatment of Personality Disorders (PDs) in the past two decades has been strongly influenced by three parallel developments. First, new expert treatment models have been established like Mentalization-Based Treatment (MBT) (Bateman et al., 2023), Dialectic Behavioral Treatment (DBT) (Linehan, 1993), Transference-Focused-Psychotherapy (TFP) (Kernberg et al., 2008) and Schema Therapy (ST) (Kellogg & Young, 2006) that by now are regarded as evidence-based (Storebø et al., 2020) and are commonly summarized as the “big 4” (Rameckers et al., 2021) bearing in mind that there are other effective treatments for PDs available. As stated in the respective treatment manuals, most of these treatments (MBT, DBT, ST, TFP) have integrated techniques from different therapeutic traditions (psychodynamic, cognitive-behavioral, humanistic and systemic) and have further expanded ideas about the developmental pathways of personality problems and how best to address them.

Second, because specialized treatments are often time- and resource-intensive, a need was identified to also establish effective therapy for PDs reflected in treatment protocols that address mental health problems related to impaired personality functioning (Hutsebaut et al., 2020). To this end, treatment approaches like Good Psychiatric Management (Choi-Kain & Sharp, 2021; Gunderson & Links, 2014) have identified the common features that make PD treatment work and have packaged these features in a generalist approach that can be used in clinical practice.

Third, in parallel to these developments, the new classification systems of DSM-5 and ICD-11 identified personality functioning as the common core of personality disorders, characterized by problems in self (identity and self-direction) and in interpersonal (empathy and intimacy) functioning. Interestingly, all “big 4” in the expert-treatments of PDs address personality functioning in general while privileging different facets of disturbed personality functioning: TFP (identity), ST (self-representation) and DBT (self-direction) focus on the functioning of the self, MBT concentrates on self with others (empathy, and self-and other understanding). All approaches work on intimacy problems by offering a secure attachment with the therapist and by working with varying degrees of directiveness and with the therapeutic relationship; from a more coach-stance in DBT to interpreting transference (enactment of dysfunctional relationship expectations) in TFP. Despite these differences, none of the “big 4” appear to be superior to another in terms of treatment effectiveness (Storebø et al., 2020). However, they have rarely been compared directly to each other and empirical proof for the exact mechanisms of change associated with each approach remains largely unknown. However, this is true for all specific and common factors in psychotherapy (Cuijpers et al., 2019). Furthermore, recent reviews on change mechanisms has revealed the non-specificity of change mechanism so that they are neither treatment-, nor disorder-specific. (Lemmens et al., 2016; Taubner et al., 2023). Strikingly, there is almost no agreement in the research field which mediators should be assessed and which measures should be used. Focusing on psychotherapy with adolescents, Taubner et al. (2023) identified 106 mediator RCTs using 252 different mediator variables (grouped in cognitive, emotional, behavioral, family, therapy or peer-related domains) that were assessed with 181 different measures. For mechanisms of change in PD, Keefe and Derubeis (2019) evaluated changes in attachment-representations, mentalization, core beliefs and defense-mechanisms as potential mediators. Only changes in defense-mechanisms obtained enough empirical support to be regarded a mediator of change in PD treatment. In a recent systematic review on mediators of change in PD treatment, Volkert et al. (2021b) identified 22 RCTS in which the majority (*k* = 15) focused on the therapeutic alliance as the most important mechanism of change. However, inconclusive results were detected for specific mechanisms, e.g. change of schemas did not explain changes in symptoms whereas changes in mentalizing, defensive functioning and use of skills explained changes at least partially (Volkert et al., 2021a, 2021b). Furthermore, mentalization appears to be a general mechanism of change in psychotherapy – not limited to the treatment of PD – based on a systematic review that included 29 studies on this question (Luyten et al., 2024).

Against this background, recent treatment developments are characterized by more modular, personalized and integrative interventions in the general field of psychotherapy (Lutz et al., 2022) calling for meta-models of general clinical change (Eubanks & Goldfried, 2019). Meta-models of change can serve the purpose of overcoming conceptual inconsistencies in traditional psychotherapy traditions (Lutz et al., 2021). Meta-models of change also provide a framework to study transtheoretical change processes if a certain agreement can be reached in the field. This is consistent with the call from Lancet Psychiatry Commission to move the field of psychotherapy to the level of mechanisms, starting with conceptual clarity, followed by experimental methods to isolate mediator candidates that should be rigorously tested in isolated treatment interventions (Holmes et al., 2018). Therefore, with this statement we will argue for a meta-model in the treatment of PDs (and psychopathology writ-large) that is transdiagnostic across PDs and transtheoretical across different therapeutic orientations. We have identified a core process of change related to mental flexibility through implicit social learning and will propose recommendations for stance and technique that are informed by research on MBT and the learning components as represented in the Mediational Intervention for Sensitizing Caregivers (MISC; Klein, 1996; Sharp & Marais, 2022; Sharp et al., 2020).

## Evidence From Developmental Psychopathology

Conclusions from longitudinal research in developmental psychopathology (Caspi et al., 2014) and large clinical samples (Fonagy et al., 2017; Sharp et al., 2015) established the idea of a general p-factor in psychopathology, meaning that instead of focusing on distinct categorical sets of mental disorders, we can model mental problems on a shared continuum of severity. Although, the p-factor model has been challenged in the field (Watts et al., 2022), we agree with Caspi et al. (2024) that these concerns may be unwarranted. Moreover, Fonagy et al. (2017) among others suggested that psychopathology can be conceptualized by the degree of absence of resilience, drawing our attention away from symptoms towards protective resources and mental capabilities or skills that evolve during childhood and adolescence. As such, developmental psychopathology serves as a strong foundation for meta-models of change in psychotherapy that shifts attention from current presentation of mental problems to etiologies of mental disorders that embrace complexity within a transactional, developmental and culturally sensitive frame. To facilitate resilience as a new goal in psychotherapy means to also shift therapeutic goals from adjustment to a certain cultural norm or definition of mental health to a more open way of creating mental flexibility in individuals. Such flexibility is conditional not only for adaptation in adult role function as adolescents age into adulthood, but also in the pursuit of wellbeing, bearing in mind constantly changing socio-political circumstances and contexts. As such, mental (or cognitive) flexibility becomes that which reduces psychopathology while enhancing resilience. This has particular relevance for personality pathology which is characterized by rigid and maladaptive patterns of relating to self-and others and an inability to flexibly respond to the stochastic nature of interactions and relationships (Sharp & Bevington, 2022; Sharp et al., 2012).

Central to the capacity for the flexible response and adaptation to a constantly changing environment is the ability to learn. Learning takes place in all kinds of contexts (including psychotherapy), the first (and arguably the most potent) of which is within the serve-and-return with primary caregivers. It is within this context that the transmission of cultural knowledge first takes place. And it is within this context that epistemic trust is established in the child – that is, the notion that learning from others is worthwhile and in a person’s best interest. Defined as “an individual’s willingness to consider communication conveying the knowledge from someone as trustworthy, generalizable and relevant to the self” (Fonagy et al., 2017, p. 766), epistemic trust develops in the context of secure attachment relationships (Harris & Corriveau, 2011). Through repeated exchanges with the caregiver, the infant or child learns that their caregiver is a trusted source of knowledge enabling learning about the self, others and the world. The mechanics of how this learning takes place is not explained by attachment theory, but rather cognitive developmental theory. Grounded in Vygotsky’s (1978) theory of social learning, Feuerstein’s (1979) theory of cognitive modifiability and Klein’s (1996) extension thereof, the mechanics of learning rely on a set of prerequisites that allows the caregiver to create a mediated learning experience (MLE) for a child. Put differently, learning is enhanced when the environment or subjective experience of the child is intentionally, actively and non-intrusively mediated for the child. While intentionality is central to creating an MLE, the learning that takes place is implicit in the sense that the caregiver is not actively teaching; rather, shared knowledge that is relevant to the unique characteristics and experiences of the child develops within the serve-and-return between caregiver and child. Elsewhere we have argued that this implicit form of learning that takes place within the serve-and-return is essential for optimal learning – whether that learning takes place in the context of the caregiver-child interaction or the interaction between psychotherapist and client (Sharp et al., 2020) – a thesis that we further elaborate here.

## Explicit Learning, Corrective Emotional Experiences and Micro-Process

Many psychotherapies use psychoeducation and explicitly link behavior with thoughts and feelings to create new knowledge and perspectives to change symptoms. Psychodynamic approaches, for example, aim for insight into one’s wishes, anxieties and defenses to find better solutions for intra- and interpersonal conflicts and use the therapeutic relationship as a stage to observe and interpret these phenomena. Therefore, psychotherapy may use explicit learning by either teaching (e.g. psychoeducation, exercise, worksheets) or by explicitly interpreting ways of behaving in relationships (e.g. transference interpretations). In contrast to specific techniques, the contextual model of psychotherapy has emphasized the role of common factors to explain variance in outcome such as therapeutic alliance, empathy, responsiveness, repairing ruptures, etc. (Norcross & Lambert, 2011; Wampold, 2015). However, the general meta-models of change as proposed by Grawe (1997) as well as Orlinsky and Howard (1987) remained too descriptive or not explaining the actual change process thereby still leaving unresolved the question as to what micro-processes between patient and therapist happen within and from session to session. Following the convincing evidence about the impact of common factors, instead of explicit learning via psychoeducation and insight, we propose to consider implicit learning with the therapist as the starting-point to understand psychotherapeutic impact. Opposed to more instructional, interpretative learning or skill-based learning, implicit change involves the facilitation of a schema for reflection, a move from content (what) to process (how). Implicit learning serves to create a mental capacity to learn how to resolve any life challenge in the future and thus leads to autonomy, agency and independence from teachers, experts and therapists to discover own solutions.

Therefore, in contrast to classic change models of explicit learning, this approach suggests implicit learning as groundwork to create new resources to adapt to life challenges. Alexander and French (1946) described the development of the psychodynamic technique from cathartic hypnosis, suggestion, free association to unlock the unconscious, working through transference neurosis until the emotional reeducation which can be seen as a meta-model of common factors and implicit learning. The authors emphasized that the classic psychoanalytic technique is to stress the repetition of the old conflict in the therapeutic relationship and to emphasize the similarity of the old conflict situation to the current transference situation. The therapeutic significance of the differences between the original conflict situation and the present therapeutic situation is often overlooked. However, it is in this difference that the value of the therapeutic procedure lies. Because the therapist's stance and role are different from that of the caregiving person of the past, the patient is given the opportunity to face again and again, under more favorable circumstances, those emotional situations which were formerly unbearable and to deal with them in a manner different from the old (Alexander & French, 1946). This idea of discriminating between the old and the new relationship to update relationship knowledge, create more mental flexibility and leave behind rigid maladaptive relationship patterns has been further developed in the control-mastery theory (Silberschatz, 2005) discriminative exercises (McCullough, 2000), limited reparenting (Kellogg & Young, 2006) and the plan-based therapeutic relationship (Caspar & Goldfried, 2018). However, the capacity to understand and process corrective emotional experiences may be limited in patients with PDs (Fonagy & Luyten, 2009). In PD treatment, clinicians are faced with a patient that appears unwilling or unable to learn from new relationships and also from the therapeutic relationship as negative expectations and low reflective functioning hinder the perception and internalization of new experiences. Furthermore, mistrust in interpersonally transmitted knowledge is highly prevalent (termed as epistemic hypervigilance). Some patients with PD may also over-identify with the therapist and the method, bearing the risk of pretend mode and credulity which does not generalize to other relationships outside the consulting room as they simply adjust to or idealize the therapist. In this case, therapists may be perceived as the better or ideal parent which can lead to further alienation within families, loyalty conflicts, devaluation of parents, parent blaming as well as a dependency on the therapist. Here, we propose that mistrust, credulity and low mentalizing within corrective emotional experiences can be helpfully addressed by using interventions and stance that rely on implicit learning such as MBT and MISC.

## Lessons Learned From MBT

Mentalization-based treatment has reconsidered the role of insight and transference in the therapeutic work with PD patients as their vulnerability in mentalizing is often triggered by the therapeutic relationship itself via attachment anxieties. Thus, the two main functions of psychotherapy, getting support and having new perspectives by a helpful professional, are severely limited in PDs. Furthermore, epistemic trust is compromised for the same reason that mentalizing and attachment fail to be a resource, based on real or perceived histories of abuse and neglect in patients with PD. To facilitate mentalizing and epistemic trust, the MBT therapist adheres to a strict not-knowing stance and adjust all interventions to the current level of the ability to reflect upon self and others. Mentalization which is related to mental flexibility is trained within the therapeutic relationship starting from mental exploration, clarification and challenging beliefs while sensitively keeping an eye on anxiety and arousal levels. If the anxiety/arousal increases, the MBT-therapist is asked to switch strategies from prompting mentalizing to supportive co-regulation and kick-starting mentalizing again by stop-and-rewind techniques as well as specific interventions for specific pre-mentalizing modes of thinking.

Mentalizing the relationship with the client is seen as a key component especially when the so-called “elephant in the room” is addressed – that is, affects in relation to the current session and the therapist. In contrast to classic psychodynamic therapies, the therapist engages in the “real” felt relationship with the patient instead of concentrating on the transference, i.e. the relationship as repetition of former relationships and tries to stay close to the patient’s current representation of the self (trying to see the world through their eyes). In so doing, the therapist discloses their own thoughts and feelings if this is helpful. In so doing, the patient learns new or other perspectives on relationships and perception of self and others, making explicit what normally stays hidden. As such, the MBT therapist models effective mentalizing and engages with curiosity and interest in the current, real therapeutic relationship with the patient, i.e. owning and actively repairing all misunderstandings, conflicts and lapses in empathy (maybe enactments) that are typical for real (authentic) relationships. All in all, this way of relating and intervening is thought to train the “mentalizing-muscle” instead of reaching a certain insight into motivations for feelings, and thus serves a more implicit corrective emotional experience. The therapeutic goal is indeed to help patients learn to mentalize effectively through implicit learning instead of mentalizing *for* them, e.g. explaining behavior to them (which would be explicit learning). However, as MBT-training is mainly acquired through experts during supervision, it was recently criticized for being too abstract, too complex and not fine-grained enough in the planning (or evaluation) of minute-by-minute interventions or micro-processes. Furthermore, sensitizing therapists to the implicit learning potential of the MBT-interactions may lead to an even stronger impact and may help therapists to better navigate the micro-processes involved. Lastly, easier programs that enable changes in mental flexibility in patients and caregivers are needed to be implemented for non-expert therapy in GPM models and for non-psychotherapeutic staff such as nurses, social workers as well as pedagogical (e.g. teachers) and early care professionals (Georg et al., 2022).

## Lessons Learned From MISC

Sharp et al. (2020) proposed that the MISC offers the very minute-to-minute micro-processes that culminate in social learning, and by extension, results in the recipient feeling mentalized. The starting point for the development of MISC was Klein’s (1996, 2001) observation that, notwithstanding significant differences between cultures, flexibility of mind and the capacity to learn from experience are evident in all cultures. Klein identified the caregiver as pivotal in creating a predisposition for learning in taking on the role of the “mediator” who is responsible for the transmission of cultural knowledge (Klein, 1996, 2001; Klein & Rye, 2004). To create a mediated learning experience (MLE), an interaction must be intentional and reciprocal, must transcend the satisfaction of an immediate need, and must focus on conveying meaning, matching it to the child’s responses.

**Figure 1:**
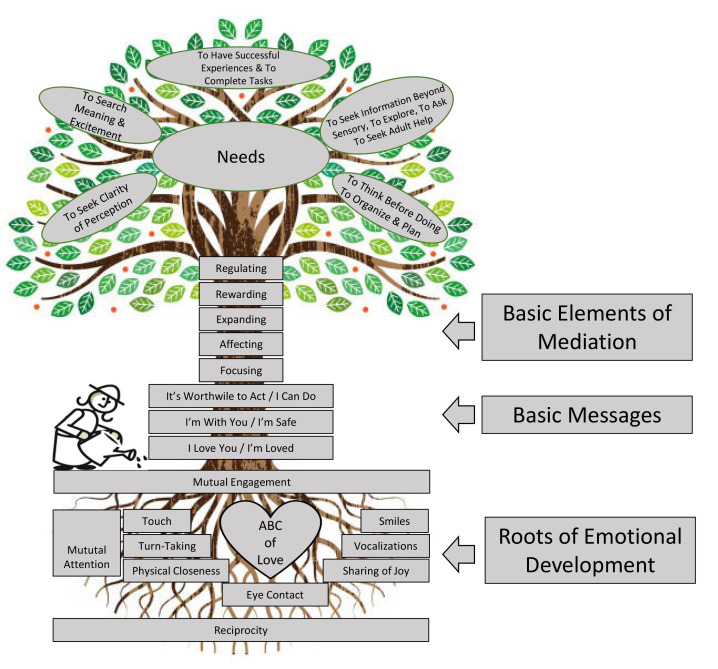
The MISC Tree *Note.* © Paina S. Klein, full copy right was granted for Carla Sharp to use this figure.

The overlap with the concept of mentalizing is clear; however, MISC extends the concept of mentalizing by describing concrete, behaviorally operationalized emotional and cognitive (learning/mediational) components that helps the caregiver take an inquiring and curious not-knowing stance slowing down the interaction to ensure mutual understanding and learning. As displayed in Figure 1 (the MISC tree), the emotional components of the MISC are the roots of facilitating learning in others. These components are already part of the relational basis of all psychotherapies and include eye contact, smiles, vocalization, touch, physical closeness, turn-taking, sharing of joy, expression of positive affect, synchrony, length of communication chains, and excitement expressed toward things, people and experiences in the environment. However, the emotional components are necessary, but not sufficient, for learning to take place. For learning to take place, cognitive components (also referred to as learning or mediational components) are necessary. These form the trunk of the MISC tree (Figure 1). Here we describe the five mediational (learning/cognitive) components while providing examples of how they would be applied in psychotherapy: *Focusing:* An act or sequence of acts that is directed toward gaining the client’s full attention (“Wait… let’s pause for a minute – this seems really important”). Through focusing, the therapist is communicating intention to teach. (2) *Providing/requesting meaning):* The therapist names, describes, and gives meaning (without interpretation) to the client’s experience (“I see you are upset”). Here, affect is important to convey additional meaning (“Wow… this is tough…. he said that he wants to leave you?”. (3) *Expanding (Transcendence):* A therapist’s behavior directed toward broadening of the client’s cognitive awareness extending the client’s understanding of what is in front of him/her by explaining, clarifying, comparing, or adding new experiences that go beyond the immediate content (“Can we just pause for a moment to unpack this a bit… it sounds very much to me like a conversation we had two weeks ago… can you remember?”). (4) *Rewarding (mediated feelings of competence with explanation)*: Any verbal or nonverbal behavior of the therapist that identifies specific components of the client’s behavior that the therapist considers successful (“You did very well in slowing down so we could talk about this in more detail….it helped me a lot to understand you better”). (5) *Regulating behavior (helping the client to plan before acting).* The therapist brings to the client’s awareness the possibility of “thinking” before doing, of planning steps of behavior toward attaining a goal by modelling, demonstrating, or scheduling events in time and space, thereby regulating the pace and reducing the client’s impulsiveness in perception, elaboration, and expression (e.g. “This is a very difficult topic to bring up with your mom… let’s first think together about how that might work out? What would be a good situation to set this up?”). As evident in the examples, the therapist is not explicitly teaching the MISC components but instead use them to slow down the interaction in service of mutual understanding. Over time, these processes are internalized and applied outside of the therapy room.

Evident in Figure 1 are also the leaves of the MISC tree. These are the outcomes for a person who was fortunate enough to experience emotional and cognitive components applied by someone interested in their wellbeing. If applied, the MISC roots and trunk stimulate an individual’s needs system – the need to seek clarity of perception, to search for meaning and excitement, to have successful experiences and complete tasks, to seek information and to think before doing – in short, agency. These too are the outcomes that we want for our clients in psychotherapy. Whereas the therapist’s role is to mediate the subjective experience for the client at the start of therapy, the end goal is for the client to foster that reflective capacity herself enabling her agency, independence and empowerment.

In summary, the MISC components represent the granular-level, behaviorally anchored, and therefore observable actions taken by the therapist to open up the epistemic highway, flexing the client’s reflective mentalizing muscles during sessions to ultimately enhance cognitive flexibility outside the therapy room. These components can be coded frame-by-frame and moment-by-moment using the Observing Mediational Interaction tool (OMI; Kerr et al., 2023; Klein, 1996), thereby operationalizing the mechanisms of change in any psychotherapy assuming that we are correct in our thesis that learning and cognitive flexibility are inherent to all effective psychotherapy. Because MISC’s evidence base is grounded in work with laypersons as MISC trainers (e.g. Bass et al., 2017; Boivin et al., 2013a, 2013b; Boivin et al., 2017; Sharp et al., 2022), its components can be learnt by paraprofessionals in healthcare thereby providing a much more scalable option to track, evaluate and teach this core and common feature of psychotherapy.

## Future Outline: Fusion of MBT and MISC

To include MISC in therapeutic processes, professionals would need to be sensitized to the emotional and cognitive components of the MISC first and learn to observe and understand their micro-interactions with their clients through video-feedback of their own sessions. The emotional components of the MISC (warmth, smiling, eye contact, synchrony, turn-taking, empathy, sharing happiness, etc.) are well in line with common factors in psychotherapy but go beyond some professional attitudes of abstinence or distance. However, in the treatment of PD emotional components alone are not strong enough to overcome epistemic mistrust. To open the gate to social learning, the client must feel understood and it is in the slowing down of the interaction through application of the mediational (cognitive/learning) components that the therapist signals a strong desire to understand the client. As explained elsewhere (Sharp et al., 2020) the mediational components powerfully cue to the recipient an interest in his/her mind, establishing a “royal road” to the formation of epistemic trust, because they necessarily involve recognition of the recipient’s subjectivity and agency, and signal an interest in collaboration and cooperation. A strong interest in the client’s mind is communicated, while giving generous access to the therapist’s mind—marking the availability of the therapist’s mind for the client’s learning, as well as the investment and interest of the therapist’s mind in the client. These components may be especially useful in high emotional interactions where therapist mentalizing shuts down, as they help to structure the interaction giving the therapist time to recover their own mentalizing. MBT is already in line with many ideas from MISC in its outline and has differentiated more clearly, as described above, that mentalizing the partner in an implicit learning interaction is the fundamental ingredient to have a sensitive teaching moment. As such, the stance of not-knowing the exact mental states of the other, being mindful of the “teacher’s” own mentalizing and staying curious without interpreting, needs to be added to the MISC intervention. Bringing both approaches together opens the possibility to implement and observe micro changes in what should be termed now implicit cognitive and emotional corrective experiences. With all modesty, we try to argue that the here outlined implicit mediated learning in the social context of a professional relationship between therapist and patient has indeed been the core change mechanism of corrective emotional experiences. However, in former descriptions of corrective emotional experiences in psychotherapy, explicit naming of and insight in differences between now and then have been the focus of elaborating this mechanism of change. Thus, we propose to shift attention and understanding towards implicit learning within professional relationships, meaning internalizing a new way of thinking about any life-event that requires adaption and thus creating adaptive capacities via mental flexibility as the general change mechanism of PD treatment in any therapeutic setting that should be investigated in the future.
